# Supplementary material for: Splice-Junction-Based Mapping of Alternative Isoforms in the Human Proteome
Source: Cell Rep. Author manuscript; Available in PMC 2020 Jan 15. (PMC6961840; doi:10.1016/j.celrep.2019.11.026)
Supplement: 2 [file NIHMS1546469-supplement-2.zip › DF1/PXD010154/Prostate/Prostate_10_FLNA_FGGEHVPNSPFQVTAPERPLVGVNGLDVTSLRPFDLVIPFTIK.pdf]

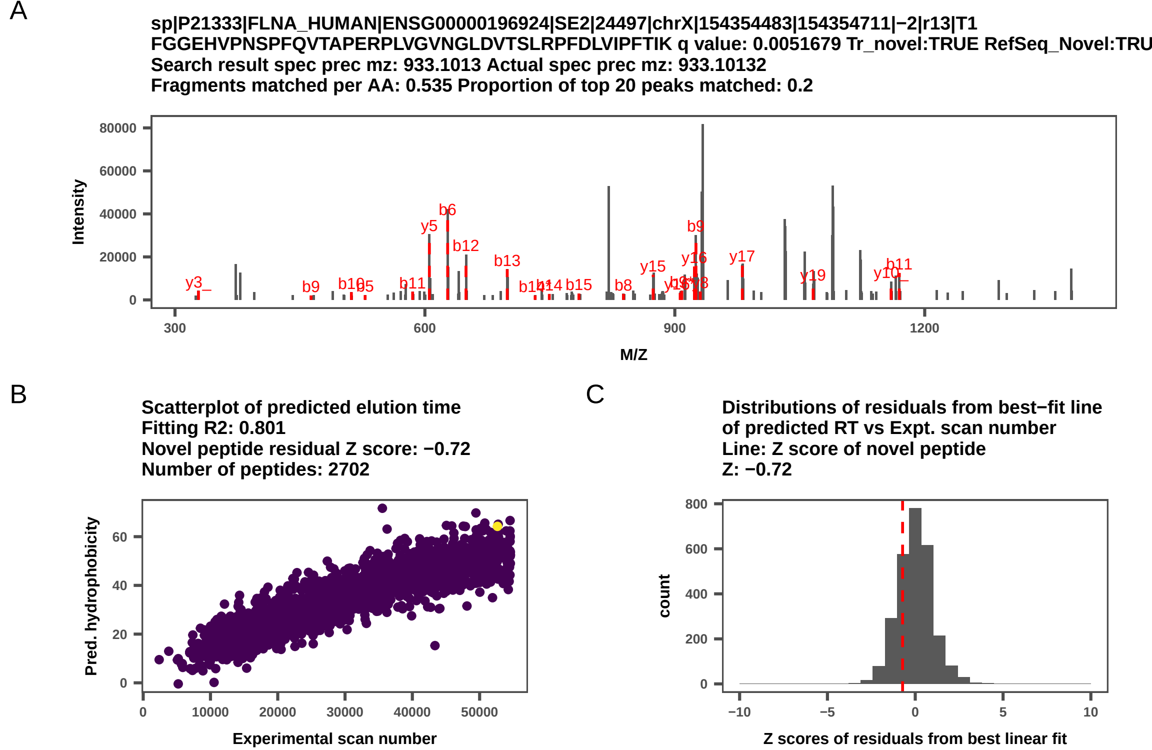
A
sp|P21333|FLNA_HUMAN|ENSG00000196924|SE2|24497|chrX|154354483|154354711|−2|r13|T1
FGGEHVPNSPFQVTAPERPLVGVNGLDVTSLRPFDLVIPFTIK q value: 0.0051679 Tr_novel:TRUE RefSeq_Novel:TRU
Search result spec prec mz: 933.1013 Actual spec prec mz: 933.10132
Fragments matched per AA: 0.535 Proportion of top 20 peaks matched: 0.2
Intensity
80000
60000
40000
20000
0
y3_
b9
y5
b6
b12
b11
b13
b15
b8
y15
b9
y16
y17
y19
b11
300
600
900
1200
M/Z
B
Scatterplot of predicted elution time
Fitting R2: 0.801
Novel peptide residual Z score: −0.72
Number of peptides: 2702
Pred. hydrophobicity
60
40
20
0
0
10000
20000
30000
40000
50000
Experimental scan number
C
Distributions of residuals from best−fit line
of predicted RT vs Expt. scan number
Line: Z score of novel peptide
Z: −0.72
count
800
600
400
200
0
−10
−5
0
5
10
Z scores of residuals from best linear fit
